# Mineralization Controls Informative Biomarker Preservation Associated With Soft Part Fossilization in Deep Time

**DOI:** 10.1111/gbi.70030

**Published:** 2025-09-18

**Authors:** Madison Tripp, Jasmina Wiemann, Luke Brosnan, William D. A. Rickard, Vivi Vajda, Michael Ernst Böttcher, Paul F. Greenwood, Kliti Grice

**Affiliations:** ^1^ Western Australian Organic and Isotope Geochemistry Centre, School of Earth and Planetary Sciences Curtin University Bentley WA Australia; ^2^ Department of Earth and Planetary Sciences Johns Hopkins University Baltimore Maryland USA; ^3^ Center for Functional Anatomy and Evolution, Johns Hopkins School of Medicine Baltimore Maryland USA; ^4^ Division of Paleontology American Museum of Natural History New York USA; ^5^ John de Laeter Centre, Curtin University Bentley WA Australia; ^6^ Department of Paleobiology, Swedish Museum of Natural History Stockholm Sweden; ^7^ Geochemistry & Isotope Biogeochemistry Leibniz Institute for Baltic Sea Research (IOW) Warnemünde Germany; ^8^ Marine Geochemistry University of Greifswald Greifswald Germany; ^9^ Interdisciplinary Faculty University of Rostock Rostock Germany

**Keywords:** biomarkers, carbonate concretions, coprolites, organic geochemistry, phosphatization, Raman spectroscopy, time of flight‐secondary ion mass spectrometry

## Abstract

Diagenetically mineralized fossil tissues represent invaluable paleobiological evidence of past life. Lipid biomarkers may be identified alongside fossils, yet the relationship between localized, diagenetic mineral precipitation, and lipid preservation remains underexplored. Coprolites (fossilized feces) attract a unique diversity of early diagenetic minerals including carbonates and phosphates, within individual samples, mediating molecular preservation of soluble lipid biomarkers alongside exceptional morphological preservation. Analysis of a well‐preserved coprolite from the Carboniferous (307 ± 0.1 Ma) Mazon Creek assemblage, USA via time of flight‐secondary ion mass spectrometry (ToF‐SIMS) spatial compound mapping demonstrated the association of 5α,14α,17α(H) 20*R* cholestane, a C_27_ dietary sterane, with iron carbonate (and some pyrite) rather than phosphate minerals. Furthermore, Raman spectroscopic fingerprinting of a suite of organic‐rich fossils spanning a number of biological species and preserved across the Mazon Creek site and other depositional settings was utilized to explore whether the localized preservation of steroids in carbonate phases represents a *lagerstätten*‐specific or generalizable pattern. Our spectroscopic analyses demonstrate a significant positive correlation between signatures of lipid biomarkers and carbonates rather than phosphates across all soft‐part samples at the Mazon Creek site and throughout Phanerozoic time and space. Early diagenetic carbonate measurably immobilizes otherwise labile lipid biomarkers and shields them against diagenetic stressors. Localized preservation identifies carbonate phases as a preferential resource for lipid‐based biological information and reveals organomineral associations as a new frontier in understanding the survival of molecules in deep time.

## 1 Introduction

Encasement in carbonate concretions, for example, calcite (calcium carbonate; CaCO_3_), siderite (iron carbonate; FeCO_3_), dolomite (calcium magnesium carbonate; CaMg(CO_3_)_2_), or rhodochrosite (manganese carbonate; MnCO_3_), can provide the necessary factors to facilitate exceptional preservation of fossils and soft tissues. Fossil preservation within carbonate concretions is dependent on numerous complex microbial, biological, sedimentological, and biogeochemical processes (e.g., Coleman and Raiswell 1981; Curtis et al. 1972; Dhami et al. 2023; Grice et al. 2019; Baird et al. 1986; Coleman 1993; McCoy 2014). The formation of concretions is mediated by bacterial processes in an inorganic sedimentary matrix (e.g., Coleman and Raiswell 1981; Curtis et al. 1972; Dhami et al. 2023; Grice et al. 2019; Irwin et al. 1977; Janssen et al. 2022; McCoy 2014; McCoy et al. 2015a; McCoy et al. 2015b), induced by the presence of decaying organic matter at the nucleus (Baird et al. 1986; Coleman 1993; Raiswell and Fisher 2000) with fossilization processes initiated before significant organism degradation can occur (e.g., Allison 1988a, 1988b; Briggs 2003). Seepage of organic matter into the surrounding pore water can yield pH conditions supporting the rapid precipitation of carbonates (i.e., weeks to months timescale; Lin et al. 2020; Yoshida et al. 2015, 2018; Zeng and Tice 2014). Lipid biomarkers (molecular fossils, e.g., cholestane) and, in extreme cases, functionalized biomolecules including cholesterol have been documented from exceptionally preserved fossils in concretions (e.g., Lengger et al. 2017; Manning et al. 2009; Melendez, Grice, and Schwark, 2013; Melendez, Grice, Trinajstic, et al. 2013; Mojarro et al. 2022; Plet et al. 2017; Tripp et al. 2022).

Understanding the evolution and behaviors of long‐extinct organisms presents a considerable challenge, often requiring interpretations based on indirect evidence. While rare, the fossil record occasionally yields exceptional morphological specimens that directly document ancient behaviors and organismal interactions, such as those between plants and insects (Azevedo‐Schmidt et al. 2022) or beetles and dinosaurs (Peñalver et al. 2023). Among these extraordinary fossils, coprolites, or fossilized feces, stand out as an invaluable source of paleobiological insight, primarily shedding light on diet, predation patterns, and even parasitic relationships of extinct animals (Chin et al. 2003; Qvarnström et al. 2019, 2024; Vajda et al. 2016). Moreover, they can preserve delicate structures, organisms, or biomarkers that are typically scarce or absent from the broader geological record. Phosphatic coprolites are associated with carnivorous fauna, as calcium and phosphorus from bones of the prey provide ions for phosphate precipitation (e.g., Chin et al. 1998; Hollocher et al. 2010). Preservation of three‐dimensional soft tissue morphology involves replacement of organic matter during earliest diagenesis by calcium phosphate, particularly in organisms with a high proportion of organically bound calcium and phosphorus (e.g., Briggs et al. 1993; Irwin et al. 1977; Schiffbauer et al. 2014). Calcium phosphate may also replace pre‐existing calcium carbonate (e.g., Gussone et al. 2020; Penrose Jr. and Shaler 1888). Phosphatization is universally known as being responsible for specimens with the most spectacular and informative soft tissue preservation (e.g., Briggs 2003; Briggs et al. 1993; Martill 1988; Schiffbauer et al. 2014). Anaerobic microbial activity drives rapid localized precipitation of calcium phosphate, which facilitates exceptional preservation (Briggs 2003; Clements et al. 2022; Sagemann et al. 1999). For example, three‐dimensional phosphatized fish are preserved within calcium carbonate concretions at the Gogo Formation in Western Australia (e.g., Long and Trinajstic 2010) and the Santana Formation in Araripe Basin, northeast Brazil (e.g., Martill 1988).

Biomarkers in geological samples have been widely investigated using traditional organic geochemical extraction and analysis approaches, which, despite yielding valuable insights, do require destruction of rare bulk material (Peters et al. (2005) and references therein). Gas chromatography–mass spectrometry (GC–MS) and liquid chromatography–mass spectrometry (LC–MS) are commonly applied analytical methods (Brocks and Summons 2014; Grice and Eiserbeck 2014; Schouten et al. 2013; Woltering et al. 2016). They provide highly specific mass spectral information of lipid biomarkers, including resolution of many of their stereoisomers, which is fundamental to interpretations of both organic matter sources and relative thermal maturity (e.g., Farrimond et al. 1998; Peters et al. 2005). However, it is desirable to complement these analyses with methods that can provide spatial correlation of organic matter and minerals within fossils. Time of flight–secondary ion mass spectrometry (ToF‐SIMS) is a surface‐sensitive analytical technique that combines both microscopy and molecular analysis at high mass and lateral resolutions, to simultaneously characterize the organic and inorganic chemical composition in situ. The ToF‐SIMS detection of biomarkers has been established in several concept studies (e.g., Guidry et al. 2000; Siljeström et al. 2009, 2010, 2013, 2017; Steele et al. 2001; Thiel et al. 2014; Thiel and Sjövall 2011), and the technique has been applied to the analysis of organic matter in geological samples and well‐preserved fossils (e.g., Colleary et al. 2015; Greenwalt et al. 2015; Greenwalt et al. 2013; Gren et al. 2017; Heingård et al. 2021; Holman et al. 2024; Lindgren et al. 2014, 2018; Schweitzer et al. 2018; Siljeström et al. 2022; Surmik et al. 2016; Toporski et al. 2002).

Raman spectroscopy is a complementary nondestructive, rapid in situ analytical technique that can be used to chemically characterize complex biological and geological materials (Butler et al. 2016; Talari et al. 2015; Wiemann et al. 2018, 2020, 2022). During fossilization, soft tissues can undergo diagenetic crosslinking of structural biomolecules to form, for example, N‐ and S‐heterocyclic polymers (Wiemann et al. 2018). These transformation products of originally informative biomolecules produce characteristic Raman signatures that can be combined with comparative statistical analyses to identify informative chemical heterogeneities across fossils (e.g., McCoy et al. 2020; Wiemann et al. 2018, 2020, 2022).

Coprolites preserved (in part) as calcium fluorophosphate (fluorapatite; Ca_5_(PO_4_)_3_F) within siderite concretions of the Mazon Creek *Lagerstätte*, USA (late Carboniferous, 307 ± 0.1 Ma; Clements et al. 2018; Cohen et al. 2013; Peppers 1996; Pfefferkorn 1979) were recently analyzed for their biomarker compositions (Tripp et al. 2022). The identity and relative abundance of steroids in coprolites can be correlated with those of animal and plant sources to provide critical information on dietary habits of ancient organisms (e.g., Gill and Bull 2012; Leeming et al. 1996; Sistiaga et al. 2014; Tripp et al. 2022; Umamaheswaran et al. 2019). For instance, the steroid distributions in these Mazon Creek coprolites were consistent with a carnivorous diet, based on an overwhelming abundance of cholesteroid biomarkers from animals (Tripp et al. 2022).

We now apply ToF‐SIMS to characterize the spatial relationship of the steroid biomarkers with different mineral phases (siderite vs. fluorapatite) in a previously studied Mazon Creek coprolite (Tripp et al. 2022). Furthermore, we utilize Raman spectroscopy to explore the scalability of observed biomarker sequestration by diagenetic minerals across fossils sampled through Phanerozoic time and space. This study aimed to examine the physical relationship of three‐dimensional fossil morphology with lipid biomarker preservation pertaining to early diagenetic mineralization processes in a unique fossilization environment.

## 2 Materials and Methods

Extractable biomarkers have been previously identified in coprolite fossils (FMNH PE52316, PE52315, PE52336) by sophisticated GC–MS (Tripp et al. 2022). To understand the role of early diagenetic mineralization in the preservation of specific dietary lipid biomarkers, a thin section of a coprolite from this study (FMNH PE52336) was analyzed by ToF‐SIMS for spatial mapping of organics and minerals simultaneously. Independently, in situ Raman spectroscopy has been used as a complementary assessment of biomarker–mineral interactions and as a high‐throughput approach that allows for the exploration of observed relationships between lipid biomarker and carbonate and phosphate mineral signatures across a larger diverse sample set (full sample list is available as a spreadsheet in Table S1).

### 2.1 Preparation of Standards for ToF‐SIMS

C_27_ 5α,14α,17α(Η) 20*R* sterane was prepared as an approximately 1 mg/mL solution in *n*‐hexane, deposited on an uncoated silicon wafer by pipetting 6–8 drops using a preannealed (500°C for 2 h) glass pipette. Wafers were precleaned by sonicating (1× methanol, 2× dichloromethane and 2× *n*‐hexane for 1 min intervals) in a preannealed glass beaker. A procedural blank consisted of subjecting a silicon wafer to these same cleaning conditions to account for any hydrocarbon signal from contamination. Analysis showed that no hydrocarbon contamination was present on the wafers and that organic fragments from biomarker standards alone were being detected.

### 2.2 Preparation of Thin Sections

Thin section preparation was performed at Microanalysis Australia. The coprolite sample was subsampled to an appropriate thin section block size (~50 × 30 × 10 mm) using a diamond trim saw lubricated with deionized water. The saw blade and cutting table were cleaned thoroughly using deionized water between samples to remove contaminants and debris. Cut thin section blocks were air‐dried at ambient temperature. This approach was utilized to minimize contamination across samples for the purposes of ToF‐SIMS analysis. The face of each prepared block was polished to an approximately 1200 grit flat surface to produce a suitable thin section billet and mounted to a frosted glass microscope slide with RenLam epoxy resin and hardener. Thin section billets were cured to the glass slides in a spring activated mounting fixture (24 h). Using a vacuum jig, the glass‐mounted billets were trimmed of excess material using a diamond trim saw lubricated with deionized water. Billet trimming yields a billet thickness of ~500–1000 μm. Automated lapping wheels with abrasive slurry feeds were used to remove excess material from the trimmed billet to a thickness of slightly over 30 μm. Sample specimen holding fixtures and vacuum jigs were utilized to ensure the production of a ground finished thin section with even thickness. The final thinning of the thin section to 30 μm and production of a polished surface was completed with three‐ and one‐micron polishing cloths.

### 2.3 ToF‐SIMS Analysis

ToF‐SIMS analyses were performed on an M6 instrument (ION‐TOF GmbH, Germany). Analysis was carried out using a high‐resolution bismuth liquid metal ion gun (LMIG). The LMIG was operated in either “high mass resolution” or “high lateral resolution” mode. High mass resolution analyses were performed using a 30 keV Bi_3_^+^ ion source with a pulsed beam current of approximately 0.25 pA over areas varying from 100 × 100 μm^2^ to 250 × 250 μm^2^. The mass analyzer was operated in “spectrometry” mode for high mass resolution analyses of C_27_ 5α,14α,17α(Η) 20*R* sterane standard, and in “all purpose” mode (high transmission) for analysis of the coprolite. High lateral resolution analyses were performed using “delayed extraction” analyzer mode and used a 30 keV Bi_3_^+^ or Bi_3_^++^ ion source, with a pulsed beam current of approximately 0.01 pA, over areas varying from 200 × 200 μm^2^ to 300 × 300 μm^2^. An electron flood gun with low energy electrons was used for charge compensation. Each analysis location was sputter cleaned in situ, using a gas cluster ion beam (GCIB) to remove surface contaminants with minimal damage to the intact molecules of the sample. The GCIB was configured either for 10 keV Ar clusters with a median of 1600 atoms raster over the region of interest for approximately 1 min, or 2 keV Ar clusters with a median of 2400 atoms raster over the region of interest for approximately 2 min, over an area between 300 × 300 μm^2^ to 500 × 500 μm^2^.

The analysis of C_27_ 5α,14α,17α(Η) 20*R* sterane in the coprolite thin section was performed in high mass resolution mode for identification, and subsequently in high lateral resolution mode for spatial distribution. These spectra were used for identification of C_27_ 5α,14α,17α(Η) 20*R* sterane secondary ion fragments in the coprolite sample by comparison of exact atomic mass units. The best yield of characteristic fragment ions occurred in positive ion acquisition. Previous literature (e.g., Toporski and Steele 2004) has also demonstrated that lipid biomarkers are best analyzed in positive ion acquisition; therefore, sample analyses here were also focused on positive ions. Spectra were calibrated for best organic fragment ion identification using the ion fragments C_5_H_5_^+^, C_5_H_7_^+^, C_7_H_7_^+^, and C_9_H_11_^+^ (or a combination of three of these depending on the abundance of each fragment). Calibration ions were kept consistent for the comparison of sample data with C_27_ 5α,14α,17α(Η) 20*R* sterane standard to ensure correct identification of hydrocarbon secondary ions. All high mass resolution analyses were performed below the static limit of 1 × 10^13^ ions/cm^2^ to maximize secondary ion yield of molecular species (Siljeström et al. 2010; Toporski and Steele 2004). All ToF‐SIMS maps are not quantitative and are representative of relative secondary ion abundances across individual analysis areas.

### 2.4 Scanning Electron Microscopy (SEM)

A Tescan Lyra3 field emission scanning electron microscope (SEM) equipped with an energy dispersive X‐ray spectrometer (EDS) was used for SEM‐EDS examinations. Electron micrographs of the sample surface were taken under backscattered electron (BSE) mode. The samples were sputter coated with a 5 nm layer of platinum to make them conductive prior to analysis. Micrographs were taken using an accelerating voltage of 20 kV. EDS analysis was performed at 20 kV, and the data were analyzed using the Oxford Instruments AZtec version 3.4 software.

### 2.5 In Situ Raman Spectroscopy

In situ Raman spectra were collected for a diversity of Mazon Creek coprolites, animal and plant body fossils, sediments, and carbonaceous body fossils from other Phanerozoic depositional settings. These spectra have been previously analyzed as part of studies with different scopes (see Table S1); however, the spectral suite has been curated and processed here to interrogate the relationship between lipid biomarker and diagenetic carbonate and phosphate signals. Samples analyzed included 3 distinct Mazon Creek coprolites (as published in Tripp et al. 2022), 2 uncatalogued coprolites, 7 Mazon Creek plant fossils, 36 Mazon Creek carbonaceous animal fossils, 28 associated Mazon Creek sediments, as well as 65 Phanerozoic carbonaceous metazoan fossils from other depositional settings (previously published in Wiemann et al. 2020, see supporting information of this publication for all specimen details). A combination of the Horiba LabRam HR800, the LabRam Evolution, and the Witec alpha‐300 spectrometers was used, each equipped with a 532 nm excitation source. Spectra were collected at 10 s exposure time and with 10 technical replications, which were automatically mean‐averaged. In order to avoid heat damage to individual samples and oversaturation of the detector, the laser power was optimized for samples and instrument set‐ups following best practices for biological spectroscopy (Butler et al. 2016). No macroscopic visual or spectroscopic evidence for heat damage was observed. All new and previously published spectra were adaptively baselined, despiked, and normalized in SpectraGryph spectroscopic software (Menges 2020) to guarantee comparability for follow‐up statistical analyses (Butler et al. 2016; Wiemann and Heck 2024). An outlier analysis was performed across all spectral fingerprints, and inliers were selected for downstream statistical assessments. To investigate if lipid biomarkers preferentially co‐occur with carbonate or phosphate minerals, normalized relative intensities were extracted for all samples at two peak positions that produce strong signals for the diversity of unaltered and diagenetically modified cholesteroids, representing the cyclic *‐CH*_*2*_‐scissoring which produces signals located at 1437 cm^−1^ and 1446 cm^−1^ Raman shift (Edwards et al. 2011). In addition, a crystal lattice phonon diagnostic for phosphates (corresponding in the Mazon Creek samples to diagenetic fluorapatite) was extracted at 956 cm^−1^; a signal diagnostic for carbonates (corresponding in the Mazon Creek samples to diagenetic siderite; other carbonate minerals found in the diverse metazoan sample set include calcite, aragonite, vaterite, and their diagenetic varieties) was extracted at 1086 cm^−1^. The combined data matrix included four extracted signals listed by taxon and subsampled into the following data packages: Mazon Creek coprolites (*n* = 6), Mazon Creek plant and animal body fossils (*n* = 43), Mazon Creek concretion matrices (*n* = 28), and carbonaceous metazoan fossils from a diversity of other depositional settings (*n* = 65). A correlation analysis was performed in PAST 3.0 (Hammer et al. 2001), and both *p* (measure of the significance of a correlation) and Pearson's r (measure of the positive or negative nature and the strength of the correlation) values were extracted for spectral correlations between normalized intensities of the two lipid biomarker signals, as well as those of the 1437 cm^−1^ signal and the two diagenetic mineral signals (sample list and localities available in Table S1).

To account for potentially informative spectral variance related to biomarker partitioning among diagenetic minerals, we also separately analyzed whole‐spectral data in the context of a 2D‐correlational map using Python.

### 2.6 Stable Isotope Gas Mass Spectrometry: Sulfur

The reduced sulfur fractions, Cr(II) reducible and acid volatile sulfide (AVS) were obtained by a two‐step acidic distillation procedure (Fossing and Jørgensen 1989; Zopfi et al. 2008). H_2_S was trapped in a zinc acetate solution and measured spectrophotometrically (Specord 40, Analytical Jena). For isotope measurements, the ZnS was converted to Ag_2_S, washed, and dried. The stable isotope composition was measured using combustion–isotope ratio monitoring mass spectrometry (Koebsch et al. 2019). Sample powders were combusted in a Thermo elemental analyzer, connected to a Thermo Finnigan MAT 253 gas mass spectrometer via a Thermo Conflo III. Measured mass ratios were calibrated to the Vienna Canyon Diablo Troilite (VCDT) scale following (Mann et al. 2009) using international sulfide and sulfate standards and are given in the conventional δ‐notation.

## 3 Results

### 3.1 Previous Work on the Mazon Creek Coprolites: Identification of Biomarkers by GC–MS and Mineral Phase by XRD

**FIGURE 1:**
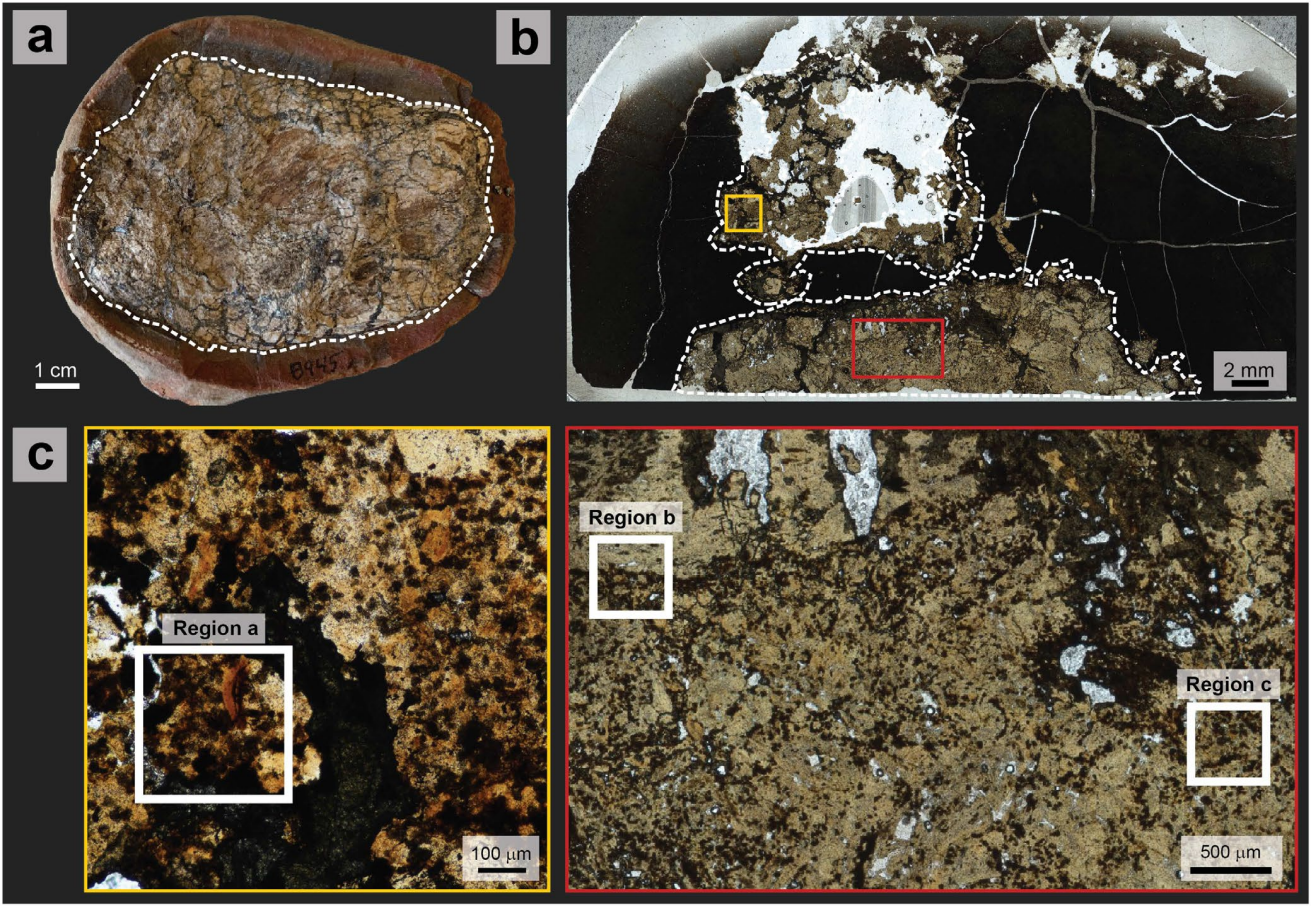
(a) Whole coprolite sample analyzed in this study (FMNH PE 52336). (b) Thin section taken through the specimen, used for ToF‐SIMS analysis. “Coprolite” region is constrained by white dashed lines. (c) Close‐up images of regions mapped using ToF‐SIMS (labelled a, b and c according to their labels in Figures 2 and 4).

The molecular composition of the coprolite analyzed in this study by ToF‐SIMS (FMNH PE52336; Figure 1) was previously characterized in detail using GC–MS and GC–MRM–MS analyses in Tripp et al. (2022), which we now summarize here. The coprolite itself was remineralized as fluorapatite (Ca_5_(PO_4_)_3_F), with veins of sphalerite (zinc sulfide, ZnS) encapsulated within an iron carbonate (siderite; FeCO_3_) concretion. The solvent‐extracted fraction of the coprolite revealed an abundance of cholesteroid biomarkers ranging from intact sterols (e.g., 5α‐cholestan‐3β‐ol) to rearranged aromatic steroids, with the most dominant being the biological isomer, cholestane, C_27_ 5α,14α,17α(Η) 20*R* sterane (5α‐cholestane). Contrastingly, the bitumen extract of the adjacent siderite matrix (concretion) showed a substantially different biomarker distribution, with predominant *n*‐alkanes and diagenetically rearranged C_27_, C_28_, and C_29_ steranes, indicating mixed organic matter sources and more extensive thermal maturation. Based on the differences in the steroid biomarker composition of the coprolite and surrounding concretion matrix, cholesteroid molecules could be confidently associated with the fossil material, and these were interpreted as evidence for a carnivorous diet of the producer animal. Preservation of such a broad suite of dietary steroids in the fossilized coprolite was attributed to the rapid remineralization of the organic material and early encapsulation within concretion‐forming siderite. Furthermore, the high abundance of fossil‐derived lipids (e.g., cholesteroids) in a multimineralized fossil made this specimen ideal for the integrated spatial mapping of organic and inorganic phases by ToF‐SIMS.

### 3.2 Identification and Spatial Mapping of Steranes by ToF‐SIMS

**FIGURE 2:**
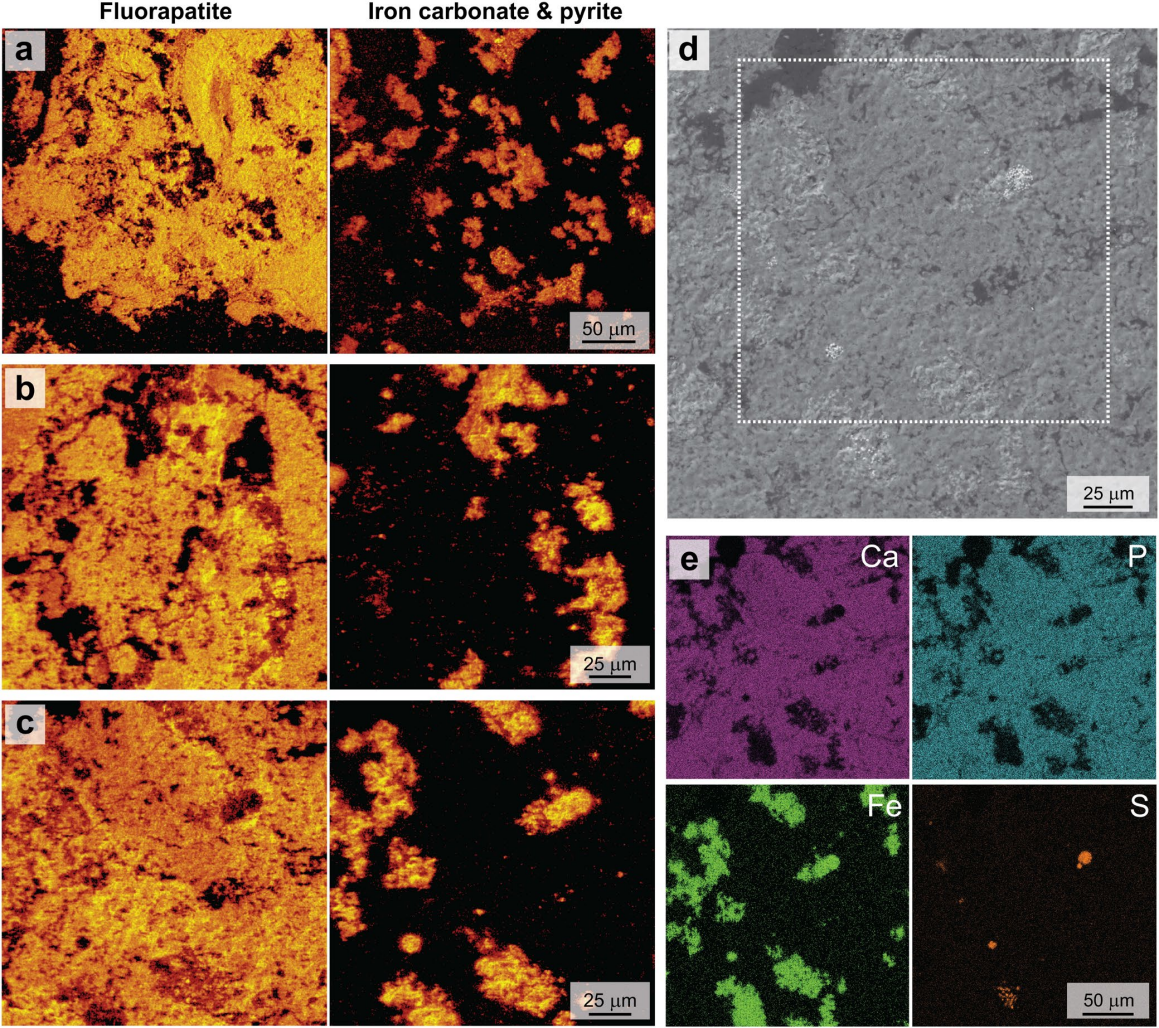
Maps from ToF‐SIMS (left) and SEM‐EDS (right) showing mineral phases in the coprolite thin section shown in Figure 1b. Left: ToF‐SIMS secondary ion maps showing relative intensities of the sum of CaF^+^ and CaPO_2_^+^ ions, representing fluorapatite, and the sum of Fe^+^ and FeH^+^ ions, representing iron carbonate and pyrite, are shown for three different coprolite regions (a, b and c). Highest relative intensities are represented by brightest regions (yellow), and maps have been scaled to increase contrast. (d) Secondary electron image of coprolite encompassing region (c), represented in the electron image by the dashed box. (e) EDS elemental distribution maps of region (d) (calcium, phosphorus, iron, and sulfur), showing distributions of fluorapatite, iron carbonate, and pyrite.

A thin section through coprolite specimen (Figure 1) was analyzed by ToF‐SIMS to investigate lipid biomarker (steroid) co‐occurrence with minerals. Main mineral phases were represented by CaF^+^ and CaPO_2_^+^ (fluorapatite) and Fe^+^ and FeH^+^ (iron minerals) (Figure 2). Although prevalent in the surrounding concretion matrix, siderite was also interspersed throughout the coprolite, which was otherwise composed of fluorapatite. EDS elemental maps of iron and sulfur abundances through the coprolite fossil region showed minor amounts of framboidal pyrite directly alongside iron carbonate (Figure 2e).

5α‐cholestane was the most dominant biomarker identified in the solvent extractable bitumen fraction of the coprolite, as identified by GC–MS (Tripp et al. 2022); therefore, it was selected to be representative of steroid content and was targeted for ToF‐SIMS mapping. A pure standard of 5α‐cholestane was analyzed by ToF‐SIMS to characterize its secondary ion mass spectrum (Figure S1), which included the molecular and deprotonated ions (i.e., M^+^, C_27_H_48_^+^ at *m/z* 372.37 and [M‐H]^+^, C_27_H_27_^+^ at *m/z* 371.36). The spectrum was dominated by low molecular weight hydrocarbon fragments (C_x_H_y_^+^, *m/z* < 110), which can also derive from other hydrocarbon biomarkers so offer limited specificity (e.g., Siljeström et al. 2009; Sjövall et al. 2008; J. Toporski and Steele 2004). Therefore, the identification and mapping of more characteristic 5α‐cholestane secondary ions (*m/z* > 110) were critical for locating organic matter indigenous to the coprolite.

**FIGURE 3:**
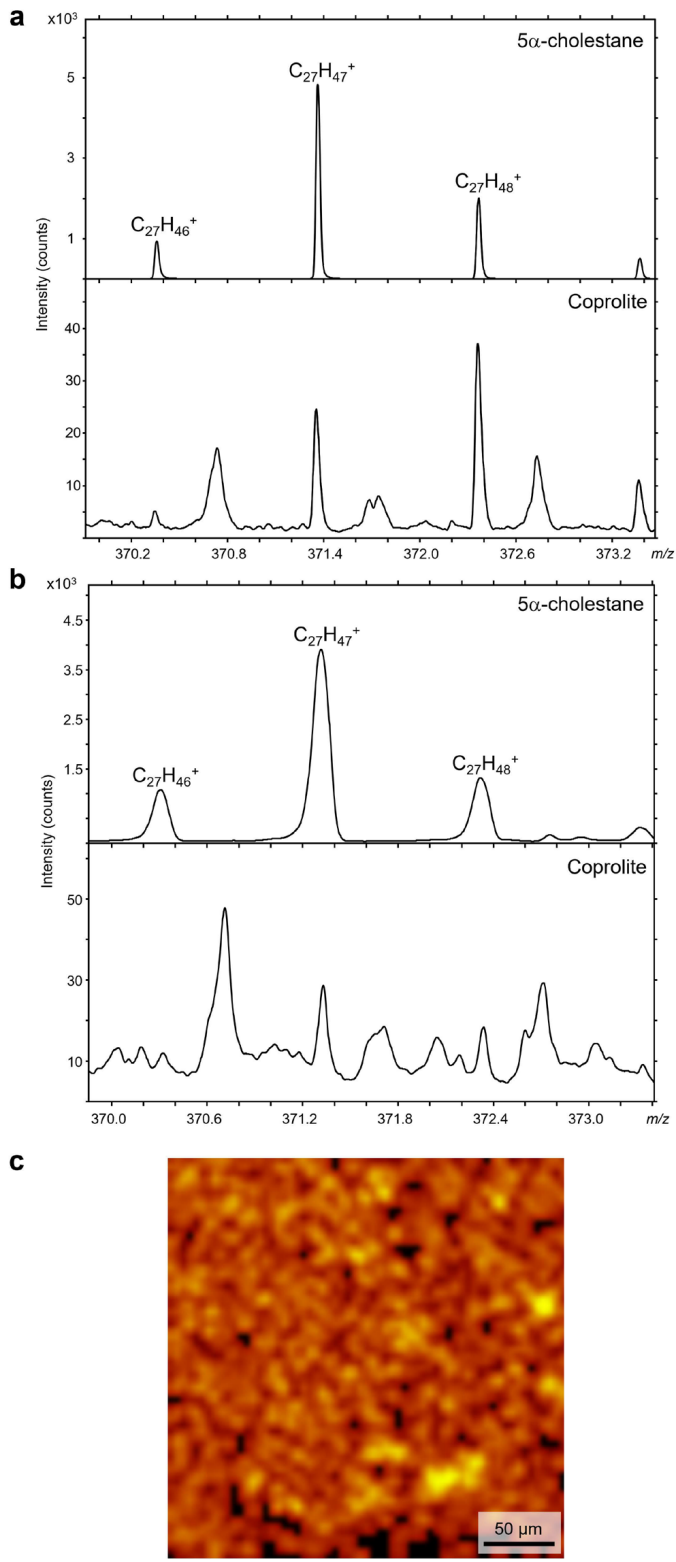
Identification of 5α‐cholestane diagnostic molecular (C_27_H_48_^+^) and deprotonated (C_27_H_47_^+^) ions in an authentic standard (top) and the coprolite fossil (bottom), taken from the same region as Figures 2a and 4a. Ions of 5α‐cholestane were identified in: (a) high mass resolution analyses; and (b) high lateral resolution analyses. The intensity of the dominant characteristic secondary ion C_27_H_47_^+^ is mapped in high lateral resolution across the fossil in (c), with an intensity filter and binned to 1024 pixels to obtain sufficient signal to identify the regions of highest intensity, which are represented by the brightest regions (yellow).

**FIGURE 4:**
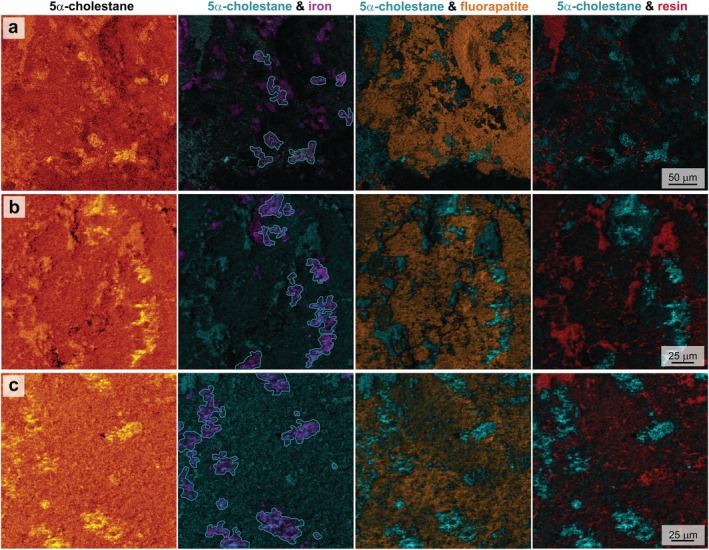
ToF‐SIMS secondary ion abundance maps from the coprolite across three regions (a, b and c as in Figure 2) of the sum of distinctive 5α‐cholestane fragment ions (a: C_6_H_9_^+^, C_7_H_11_^+^, C_8_H_13_^+^, C_11_H_17_^+^, C_16_H_25_^+^, C_27_H_47_^+^, C_27_H_48_^+^; b and c: C_6_H_9_^+^, C_7_H_11_^+^, C_8_H_13_^+^ and C_11_H_17_^+^). The individual secondary ion map of C_27_H_47_^+^ from region (a) is shown in Figure 3c. In secondary ion abundance maps of 5α‐cholestane for each region (left), the brightest regions represent highest total secondary ion signal. Organic 5α‐cholestane maps are overlaid (in blue) with maps of iron carbonate and pyrite (middle; purple), and fluorapatite (middle; orange) (secondary ion intensity maps shown in Figure 2). Blue outlined areas in 5α‐cholestane and iron maps (middle) represent regions of highest intensity of 5α‐cholestane fragment ions co‐occurring with iron phases, highlighting the overlap with siderite and pyrite. On the right is shown the overlaid organic 5α‐cholestane maps (blue) with resin maps (red) (sum of ions C_6_H_6_O^+^, C_6_H_7_O^+^, C_7_H_7_O^+^, C_9_H_11_O^+^; total abundance maps and overlay with minerals shown in Figure S3), highlighting that there is minimal overlap in the distributions of coprolite organic signals and those from resins.

Molecular and deprotonated ions (C_27_H_47_^+^, C_27_H_48_^+^) of 5α‐cholestane were identified in the coprolite at high mass resolution (m/Δm > 7000) by comparison of exact fragment masses with those of the pure standard secondary ion spectrum (Table 1; Figure 3a), as well as in high lateral resolution analyses (Figure 3b). Importantly, higher molecular weight ions representative of the molecular and deprotonated ions of ergostane (C_28_H_50_) or stigmastane (C_29_H_52_) were not present. A group of less specific, but more abundant secondary ions (C_6_H_9_^+^, C_7_H_11_^+^, C_8_H_13_^+^, C_11_H_17_^+^, C_16_H_29_^+^) was identified (Figure S2) and when mapped in high lateral resolution mode, were shown to be primarily colocalized with siderite, with minimal spatial overlap with fluorapatite domains (Figure 4). Due to the relatively weak signal of the molecular ion fragments of cholestane (both in the isolated spectrum of cholestane as compared to lower molecular weight fragments (Figure S1), and in the total secondary ion spectrum of the coprolite as compared to lower molecular weight organic fragments and inorganic fragments), these were not able to be mapped in all regions (e.g., Figure 4b,c). However, by mapping only fragments from C_6_H_9_^+^ and upward to C_11_H_17_^+^, a clear distinction from the embedding resin and association with iron phases was able to be identified. This was supported by mapping of the characteristic deprotonated ion, C_27_H_47_^+^ (Figure 3c), which was heterogeneously distributed, with regions of highest relative abundance (brightest yellow) associated with the distribution of C_6_H_9_^+^ to C_16_H_25_^+^ fragments in the same region (Figure 4a). Secondary ion maps of biomarkers in the multimineralized coprolite specimen, therefore, demonstrate that coprolite‐derived organic matter (lipid biomarkers) is intimately associated with siderite (and some pyrite) and not fluorapatite.

**TABLE 1:** Characteristic and distinctive secondary ions of C_27_ 5α,14α,17α(Η) 20*R* sterane detected by ToF‐SIMS in the coprolite at high mass resolution.

Ion assignment	Expected mass	Detected mass
C_6_H_9_^+^	81.070	81.070
C_7_H_11_^+^	95.086	95.086
C_8_H_13_^+^	109.102	109.102
C_11_H_17_^+^	149.133	149.132
C_16_H_25_^+^	217.196	217.190
C_27_H_47_^+^	371.367	371.358
C_27_H_48_^+^	372.375	372.368

To eliminate contributions from contamination during sample preparation, a thorough analysis of the embedding resin was undertaken. A range of organic fragments, including hydrocarbons and heteroatomic organic fragments (e.g., oxygen), was detected. Comparison of the resin spectrum with characteristic secondary ions of epoxy (using the IONTOF spectra library) was used to select ions that were not common to 5α‐cholestane (C_6_H_6_O^+^, C_6_H_7_O^+^, C_7_H_7_O^+^ and C_9_H_11_O^+^). These ions were selected to represent the resin in ion abundance maps (Figure 4, Figure S3) and appear as infill in voids between minerals as would be expected from the embedding material. Total ion abundance maps of organic ions from the resin (Figure S3) showed a different distribution to that of characteristic 5α‐cholestane fragments (Figure 4).

### 3.3 Early Diagenetic Mobility of Hydrocarbons Prior to Fixation in Diagenetic Carbonates

**FIGURE 5:**
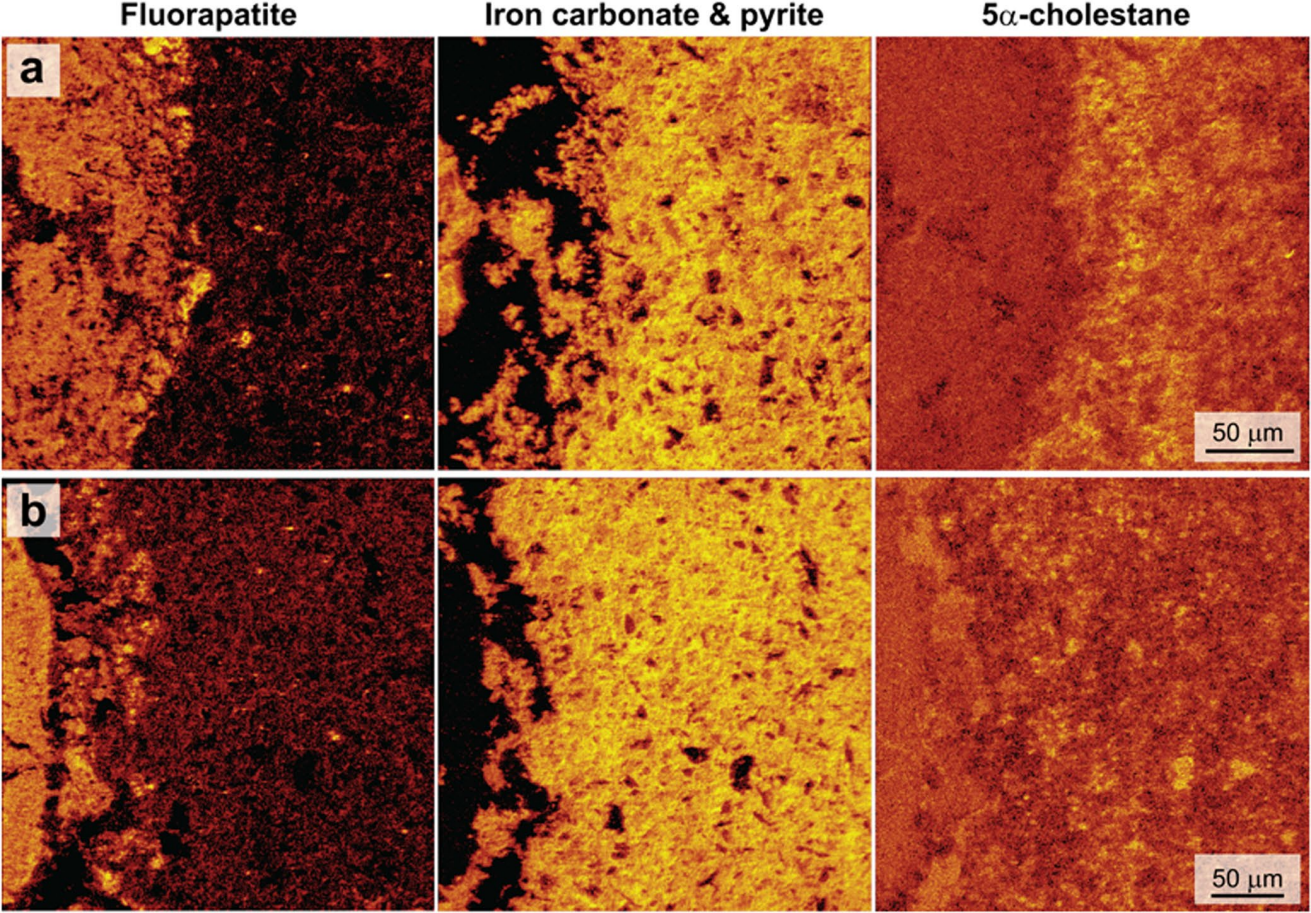
ToF‐SIMS secondary ion maps showing relative intensities of the sum of CaF^+^ and CaPO_2_^+^ ions, representing fluorapatite (left), and the sum of Fe^+^ and FeH^+^ ions, representing iron carbonate and pyrite (middle), are shown for two different regions at the interface between the coprolite and the concretion matrix (a and b). Brightest areas in fluorapatite maps (left) represent the coprolite; brightest areas in the iron maps (middle) represent the carbonate concretion. The sum of select biomarker‐derived organic fragment ions (C_6_H_9_^+^, C_7_H_11_^+^, C_8_H_13_^+^), as identified in 5α‐cholestane, are mapped on the far right.

The concretion matrix immediately surrounding the coprolite was also targeted using ToF‐SIMS to investigate the distribution of hydrocarbons through the surrounding siderite. Characteristic fragments of 5α‐cholestane were not sufficiently abundant in the surrounding matrix; therefore, less specific hydrocarbon fragments were targeted. A group of secondary ions (C_6_H_9_^+^, C_7_H_11_^+^, C_8_H_13_^+^) from hydrocarbons was shown to be elevated at the interface of the coprolite's outer edge and siderite concretion (Figure 5). This suggests that lipids were preserved in proximity to the coprolite fossil, possibly associated with the outward migration of hydrocarbons from the decaying fecal material during the early stages of carbonate concretion formation.

### 3.4 Diagenetic Carbonate, Phosphate, and Sulfide Minerals Formed During Early Diagenesis

The δ^34^S of pyrite (Chromium Reduced Sulfur, CRS, essentially pyrite) and other sulfide minerals (e.g., sphalerite, ZnS; measured as acid volatile sulfur, AVS) (Table S2) measured in two of the Mazon Creek coprolites ranged from 7.6‰ to 13.2‰. These values are quite close to the late carboniferous seawater sulfate δ^34^S value of about 15‰ vs. Vienna Canyon Diablo Troilite (VCDT) (Wu et al. 2014). In an open system, the reduced sulfur pool from sulfate reduction would be expected to be −5‰ or lower. Sulfate reduction is typically characterized by significant isotopic depletion (typically in the range −20‰ to −45‰ [e.g., Habicht and Canfield 1997; Hartmann and Nielsen 2012]). Sulfide values that are significantly more enriched (> 10‰) than anticipated reduced sulfur values (≤ −5‰) are indicative of a sulfate‐limited system where the sulfate reducers have less opportunity to preferentially utilize ^32^S. Sulfate is usually limited in deltaic environments (Berner 1981; Curtis et al. 1986), so the subsequently limited pool of reduced H_2_S may be quickly consumed by Fe^2+^ to form pyrite (FeS_2_), with minimal sulfur fractionation. Exhaustion of sulfate can result in the biochemical system then shifting to methanogenesis, which, given the same local pH environment, would mediate the reaction of residual iron with carbonate to form siderite (e.g., Cotroneo et al. 2016). Previously, high δ^13^C values of siderite in Mazon Creek concretions have been attributed to carbonate formed via methanogenesis (Cotroneo et al. 2016). Here the depleted δ^13^C value measured for the hydrocarbon biomarker phytane as compared to *n*‐alkanes, in coprolites from Mazon Creek (Tripp et al. 2022) can be attributed to a methane cycle which is typically prominent in deltaic paleoenvironments such as Mazon Creek.

### 3.5 Scaling the Observation: In Situ Raman Fingerprints Reveal a Significant Correlation of Steranes and Carbonates in Mazon Creek Coprolites and Across a Diverse Fossil Sample

**FIGURE 6:**
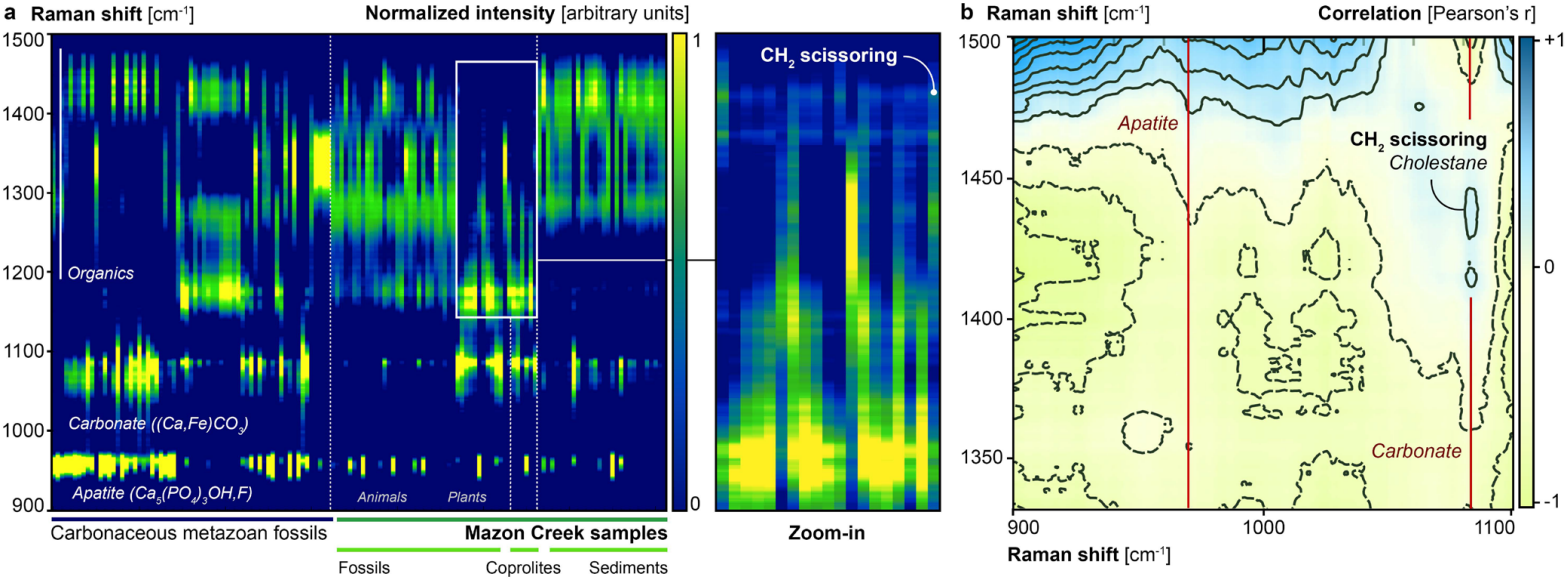
Signal distribution and analysis of Raman whole‐spectral data for Mazon Creek coprolites and other reference samples from diverse depositional settings. Raman signal distributions for the entire data set were plotted over the relevant part of the organic fingerprint region ranging from 900 to 1500 cm^−1^ (shown in a). Observed mineral bands and the region containing primarily organic signals associated with steranes are labelled, and individual sample groups are grouped together. For Mazon Creek samples, a 2D‐spectral correlation map (projecting Pearson's r) was plotted to assess the ranges of mineral signal and sterane signal correlations (shown in b). Red lines indicate the band position of crystal lattice vibrations in carbonates and phosphates, and the twin peaks diagnostic of steranes (Edwards et al. 2011) is outlined and labelled.

To assess the relationship between carbonate minerals and sterane biomarkers identified in ToF‐SIMS, Raman spectra were obtained from a range of fossils with different diagenetic mineral phases to identify relationships between lipid fingerprints and minerals (Figure 6a). Correlation analysis of Raman spectral data revealed a significant and strong positive correlation of the two Raman bands indicative of lipid biomarker cyclic *‐CH*_*2*_ scissoring, supporting a mechanistic connection between these two spectral signatures (compare Edwards et al. 2011). In the Mazon Creek coprolites, spectral features associated with lipid biomarkers were significantly and positively correlated with siderite (Pearson's *r* = 0.82 and *p* < 0.05 in Table 2; see Figure 6b), but no significant correlation with apatite was detected (see Table 2 and caption for details of representative Raman signals). Similarly, significant and positive correlations between carbonate phases and steranes were found for the Mazon Creek animal and plant body fossils—notably the observed correlations between biomarker features and diagenetic minerals were weaker (Pearson's *r* = 0.42 and *p* < 0.05 in Table 2). In Mazon Creek concretion matrices, no significant correlation was found between signals associated with biomarkers and diagenetic phosphate or carbonate (see *p* < 0.05; see Figure 6b). When analyzing biomarker signal correlations with diagenetic carbonates and phosphates in Phanerozoic metazoan samples from a diversity of localities (see Wiemann et al. 2020 and the associated supporting information for full specimen details), no significant correlation was found with diagenetic phosphate; yet, the analysis reveals a significant positive correlation with diagenetic carbonates (see Pearson's *r* = 0.39 and *p* < 0.05).

**TABLE 2:** Summary statistics of correlation analyses performed for subsets of in situ Raman spectra.

Subsample	Number of samples	Mineral	Pearson's r	*p* value
**Mazon Creek fossils**	43	Phosphate	0.15336	0.32616
		**Carbonate**	0.42833	**0.004164**
**Mazon Creek coprolites**	6	Phosphate	0.76867	0.074079
		**Carbonate**	0.81653	**0.047404**
**Mazon Creek concretion matrix**	28	Phosphate	−0.13853	0.48204
		Carbonate	0.35524	0.063581
**Diverse Metazoan fossils**	65	Phosphate	0.20752	0.097179
		**Carbonate**	0.39349	**0.001184**

*Note:* The underlaying data capture biomarker‐related organic functional group signals and crystal lattice phonons of diagenetic carbonate and phosphate minerals. The correlated spectral features associated with lipid biomarkers were selected for compound inclusivity and signal strength and include 1437 cm^−1^ and 1446 cm^−1^ (cyclic *‐CH*_*2*_ scissoring, Edwards et al. 2011), while the band diagnostic for carbonates is positioned at 1086 cm^−1^, and the signal diagnostic for phosphates is located at 956 cm^−1^. Pearson's r is listed as an indicator for the nature (positive correlation: Highlighted by blue shading; negative correlation: Red shading) and strength of the observed correlation; *p* values < 0.05 suggest a significant correlation and are highlighted in bold font, while *p* values > 0.05 indicate an insignificant correlation and are marked by grey shading (corr: Between normalized intensities at 1437 cm^−1^ and 956 cm^−1^ or 1086 cm^−1^). See Supporting Material for table of specimen details; specimen details for fossil metazoans can be found in Wiemann et al. (2020).

## 4 Discussion

### 4.1 Early Diagenetic Mineralization Processes

The remineralization of soft tissue as phosphate rather than carbonate is dependent on local pH conditions and available pore water ion concentrations (e.g., Briggs and Kear 1993, 1994; Keenan and Engel 2017; Sagemann et al. 1999). This is referred to as the “calcium carbonate‐calcium phosphate shift,” with phosphate mineral precipitation occurring where bicarbonate ion concentrations are limited and pH is lower than the dissolution pH of carbonate (approx. 6.4; Briggs and Wilby 1996), or when phosphate concentrations are high (Gussone et al. 2020). Mineral precipitation is dependent on the paleodepositional setting. In anoxic marine environments with abundant sulfate and reactive iron (Fe^2+^), biogenic sulfide reacts with the iron to precipitate pyrite, while carbonate produced upon dissimilative sulfate reduction reacts with dissolved calcium ions to precipitate calcite (e.g., Akam et al. 2020; Ben‐Yaakov 1973; Berner 1985; Curtis et al. 1986; Pye et al. 1990). Comparatively, in freshwater or deltaic settings where sulfate is limited, reduced sulfide will be rapidly consumed in pyrite formation given sufficient iron availability. With the exhaustion/absence of sulfide oxidizers in anoxic settings, methanogenesis is the dominant pathway of organic matter oxidation, and carbonate ions may instead react with iron to form siderite (e.g., Berner 1981; Curtis et al. 1986; Pye et al. 1990). This is observed in the freshwater Mazon Creek depositional setting, which is known for its siderite concretions sometimes containing minimal amounts of pyrite (Baird 1979; Baird et al. 1986). Oxygen was limited by rapid burial, with reduction of available Fe^3+^ to Fe^2+^ occurring until all Fe^3+^ was exhausted, followed by sulfate reduction—although the two can also occur simultaneously (e.g., Cotroneo et al. 2016; McCoy et al. 2017). The stable sulfur isotopic values of pyrite in Mazon Creek concretions are consistent with a transition from iron reduction to methanogenesis, and consequently, siderite precipitation probably occurred rapidly at shallow depth (Table S2; Cotroneo et al. 2016). Early microbial decay of deposited organisms produces fatty acids, lowering the pH local to the organic matter source and promoting carbonate mineral precipitation, resulting in a “proto‐concretion” that is later cemented (e.g., Bojanowski and Clarkson 2012; Cotroneo et al. 2016; Yoshida et al. 2015, 2018), during methanogenesis (Cotroneo et al. 2016) This process occurs within weeks of organic deposition (e.g., Allison 1988a; Berner 1968; Briggs and Kear 1993; McCoy et al. 2015b). The presence of exceptionally well‐preserved soft‐tissue fossils within these concretions implies the process is extremely rapid, occurring prior to, and protecting the organism from, extensive diagenetic alteration (Allison 1988a, 1988b; Briggs 2003), also demonstrated with isotopic data (Cotroneo et al. 2016; McCoy et al. 2017).

The sample in this study is an example of a complex mineralization environment, where rapid shifts in porewater chemistry have favored microenvironmental precipitation of different mineral species (e.g., Clements et al. 2018; McNamara et al. 2009; Muscente et al. 2017). The remineralization of the three‐dimensional coprolite material as fluorapatite is typical of carnivorous coprolites (e.g., Chin et al. 1998; Hollocher et al. 2005, 2010), where autochthonous calcium phosphate replaces the original tissues (e.g., Bajdek et al. 2014). Small regions of iron minerals within the three‐dimensional phosphate structure (Figure 2) support that the shift from a phosphate‐forming to a carbonate‐forming chemical environment occurred very rapidly (Briggs and Wilby 1996), at the interface of the fecal material and surrounding pore water. Moreover, the presence of minor amounts of pyrite alongside siderite in these interstitial spaces is a product of the rapid change from a sulfate‐reducing to a methanogenic environment even without burial (Cotroneo et al. 2016).

### 4.2 Implications for Understanding Lipid Preservation in Multi‐Mineralized Samples

An ongoing focal point of ToF‐SIMS studies of sedimentary organic matter is the correlation of organic components with structural features at micron scales (e.g., Toporski and Steele 2004). The colocalization of dietary biomarker secondary ion fragments with iron minerals and not fluorapatite in a phosphatized coprolite has important implications for understanding the role of early diagenetic mineralization processes in lipid biomarker preservation. Although coprolites themselves are not always strictly thought to fall within the definition of “soft tissues,” early diagenetic mineralization as fluorapatite is a process commonly associated with rapid soft tissue preservation. Therefore, the data here more broadly reflect the relationships between molecular and morphological tissue preservation. Earliest diagenetic phosphatization, while ideal for replacement and preservation of soft tissues, here does not show any relationship to biomarker preservation. Biomarker secondary ion colocalization with iron phases, predominantly iron carbonate but also minor amounts of pyrite, suggests that these minerals have facilitated molecular preservation in close association with the coprolite. Carbonate concretions can be associated with exceptional fossil preservation (e.g., Allison and Pye 1994; Grice et al. 2019; McCoy 2014), attributed to their rapid growth and the low permeability of their fine‐grained cement form (Allison 1988a; McCoy et al. 2015b), and have been demonstrated to contain source‐specific biomarkers (e.g., Manning et al. 2009; Melendez, Grice, and Schwark, 2013; Melendez, Grice, Trinajstic, et al. 2013; Mojarro et al. 2022; Plet et al. 2017; Tripp et al. 2022, 2023). The mineralization process hinders the access of degrading microbial enzymes to the biomolecules of the deceased organism (e.g., Anderson 2023; Briggs 2003; Trueman and Martill 2002) and also supports preservation via crosslinking with oxidation products (Schweitzer et al. 2014; Wiemann et al. 2018), or associated with mineral matrices (e.g., Keil et al. 1994; Salmon et al. 2000; Trueman and Martill 2002). A previously observed association of soft tissues with iron oxide particles identified iron‐mediated radical crosslinking as a tangible pathway to soft tissue preservation (Schweitzer et al. 2014; Wiemann et al. 2020). However, the dominant iron phase in the concretion in this study was siderite (as identified by XRD; Tripp et al. 2022), with iron oxide minerals occurring only at the weathered rims of the concretions (e.g., observed by Cotroneo et al. 2016). Framboidal pyrite would have formed during very early sulfate reduction, which may have also helped to preserve organic matter via rapid entombment (e.g., Baumgartner et al. 2019). This might account for the association of hydrocarbon secondary ions with framboidal pyrite (Figure 4). It is likely that the primary process responsible for lipid biomarker preservation in the coprolite sample was rapid precipitation of low porosity carbonate cement, preventing extensive migration of endogenous lipids away from their source, inhibiting the rate of diagenetic alteration of these lipids.

### 4.3 Encapsulation and Fossilization

**FIGURE 7:**
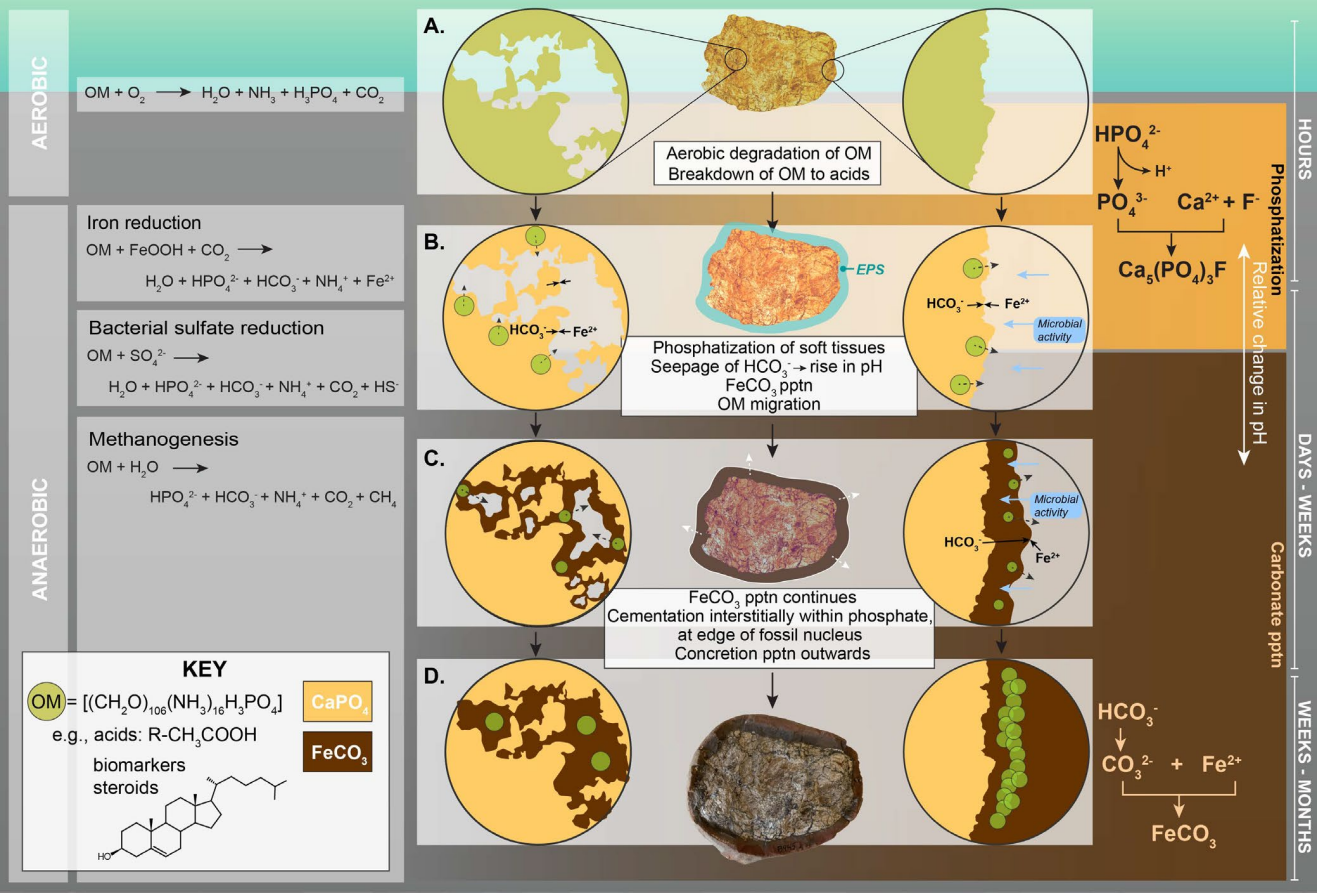
Simplified conceptual model demonstrating the pathways of bacterial organic matter degradation, chemical reactions (after Muscente et al. 2017), and mineral precipitation involved in organic matter preservation within an iron carbonate (siderite) concretion containing a phosphatized coprolite from Mazon Creek. Chemical reactions are shown both interstitially between the phosphatized soft tissue (left of sample image) (< 50 μm scale), and around the edge of the coprolite (right of sample image) forming the concretion. (a) Initial aerobic degradation of egested fecal material; (b) Rapid phosphatization of soft tissues and initial siderite precipitation; (c) Siderite precipitation forming early diagenetic cemented carbonate around the coprolite; (d) Siderite continues to form the concretion over longer time scales, and cementation helps to preserve biomarkers (steroids). OM, organic matter; pptn, precipitation.

Our study provides new insights into the series of processes responsible for the rapid encapsulation and fossilization of egested fecal material, depicted in Figure 7. The initial stages of preservation are facilitated by rapid microbial mineralization, largely driven by anaerobes. Initial degradation of the fecal material in the water column and at the sediment–water interface would lead to the release of components such as acids, sterols, or acidic byproducts from soft tissues (e.g., CO_2_, H_2_S), and phosphorus from digested bones (Figure 7a). With sediment accumulation, anaerobic processes begin to drive further degradation involving iron reduction, bacterial sulfate reduction, and methanogenesis. Microbes and extracellular polymeric substances (EPS) would develop a biofilm (e.g., Summons et al. 2013), and a “closed” system would develop. The release of acidic components would have lowered the pH which, combined with elevated phosphate in pore waters contributed by the oxidation of organic matter, may have favored rapid fluorapatite precipitation, rather than precipitation of carbonate which occurs at higher pH (Figure 7b) (Briggs and Wilby 1996). This probably occurred within hours to days following deposition. Subsequently, bicarbonate ions (HCO_3_^−^) formed from fatty acid decomposition would have raised the pH and thereby controlled the precipitation and growth of carbonate minerals (e.g., Bojanowski and Clarkson 2012; Cotroneo et al. 2016; Yoshida et al. 2015). Rapid exhaustion of sulfate, via pyritization of reduced sulfide, promoted methanogenic conditions as indicated by both δ^34^S and δ^13^C values, creating conditions favorable for siderite precipitation (e.g., Cotroneo et al. 2016). HCO_3_^−^ seeping outward from the organic nucleus formed carbonate ions (CO_3_^2−^), then reacted with Fe^2+^ in the pore water to precipitate siderite (Figure 7c). Biomarkers intimately associated with interstitial siderite and the coprolite–matrix interface (Figure 2c,d) are consistent with the migration of hydrocarbons such as steroids from the decaying fecal material during the first stages of concretion formation. Raman signals associated with biomarkers revealed a strong, positive correlation with diagenetic carbonates (Table 2) across fossil and coprolite samples, suggesting preferential preservation of these soluble biomarkers in siderite, calcite, and comparable authigenic minerals. Continuing siderite concretion growth and cementation (Figure 6d) subsequently prevented further migration (e.g., Allison 1988a) and extensive diagenetic rearrangement of steroids, demonstrated by intact sterols and thermally immature steroid configurations.

## 5 Conclusions

Biomarker colocalization with siderite and some pyrite but not fluorapatite reveals new insights into the mechanisms involved in fossil preservation when phosphate and iron are readily available in the porewaters. The evidence of precipitation and cementation of early diagenetic carbonate as a means of immobilizing lipid biomarkers and inhibiting the effects of diagenetic processes represents an important development in understanding the preservation of organic matter associated with decaying organisms. These processes have broader implications for the locality of organic molecules in other soft tissue fossils, suggesting that carbonate phases represent an important target for molecular preservation. Fluorapatite forms at a relatively early stage of diagenetic mineralization, and there are numerous reports of 3‐D soft tissue fossilization in fluorapatite minerals. Here, however, we find no preservation of dietary lipids in the fluorapatite moiety of the coprolite. The intimate association of lipid biomarker signals with the siderite phases of the concretion identifies siderite as an important host for the sequestration of early diagenetic organic molecules over long geological times. The excellent molecular preservation properties of siderite cement likely relate to its rapid rate of formation and low porosity, particularly pertaining to concretion growth around an initiating organic nucleus. Fossilization mechanisms may be dependent on redox conditions, porewater chemistry (e.g., concentrations of P^5−^, F^−^, Ca^2+^, Fe^2+^) combined with the original composition of the decaying organic material. Extending current knowledge on the role of minerals in lipid preservation is critical for targeting informative organic signals through deepest time, to provide new biomarker information of ancient organisms to further our understanding of the evolution of life.

## Conflicts of Interest

The authors declare no conflicts of interest.

## Supporting information


**Data S1:** gbi70030‐sup‐0001‐supinfo.zip.

## Data Availability

The data that support the findings of this study are available from the corresponding author upon reasonable request.
