# Supplementary material for: Mineralization Controls Informative Biomarker Preservation Associated With Soft Part Fossilization in Deep Time
Source: Geobiology. 2025 Sep 18;23(5):e70030. doi: 10.1111/gbi.70030 (PMC12446897; doi:10.1111/gbi.70030)
Supplement: Supplementary file 1 — Data S1: gbi70030‐sup‐0001‐supinfo.zip. [file GBI-23-e70030-s001.zip › gbi70030-sup-0001-supinfo.pdf]

Supporting Information for:

Mineralization controls informative biomarker preservation associated with soft part fossilization in deep time

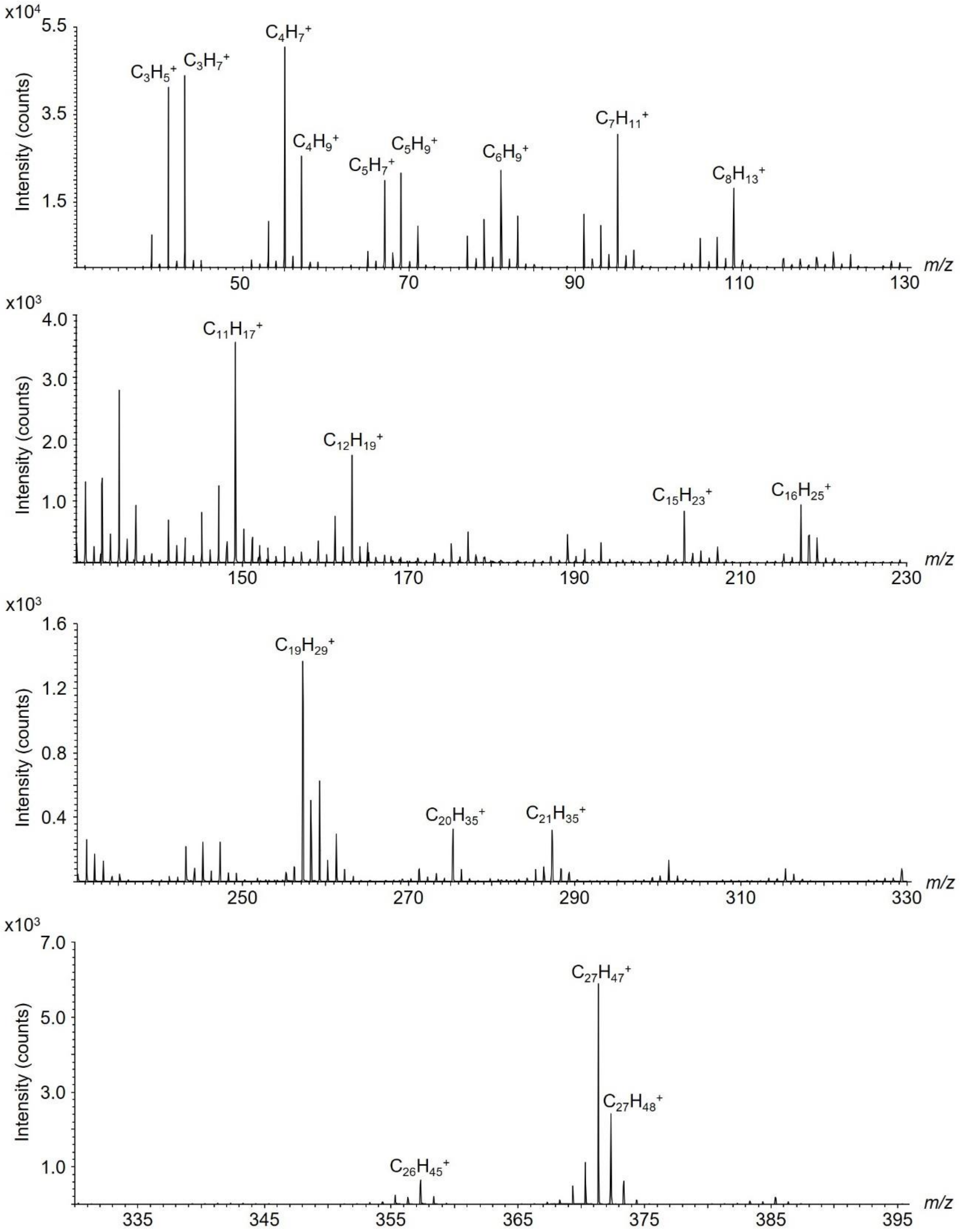

Figure S1. Positive ToF-SIMS spectra of pure cholestane standard deposited on a silicon wafer, analyzed in spectrometry mode. Selected peaks are labelled, allocated based on *m/z* values and confirmation with spectral database.

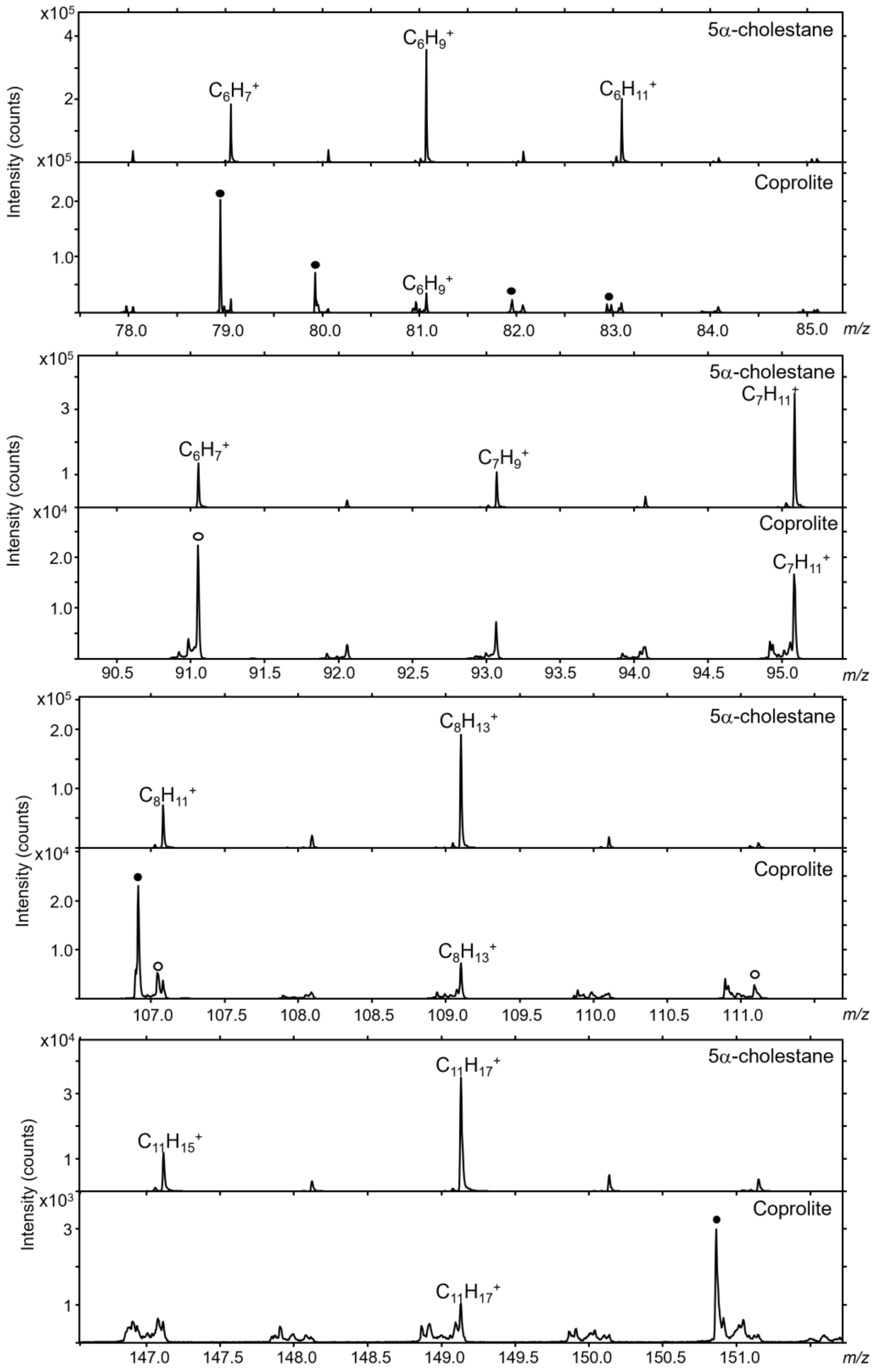

Figure S2. Identification of 5α-cholestane secondary ions in an authentic standard (top of each panel) and the coprolite fossil (bottom of each panel) in high mass resolution analyses. Selected peaks are labelled, allocated based on *m/z* values and confirmation with spectral database. Filled circles represent inorganic fragments; unfilled circles represent resin organic fragments.

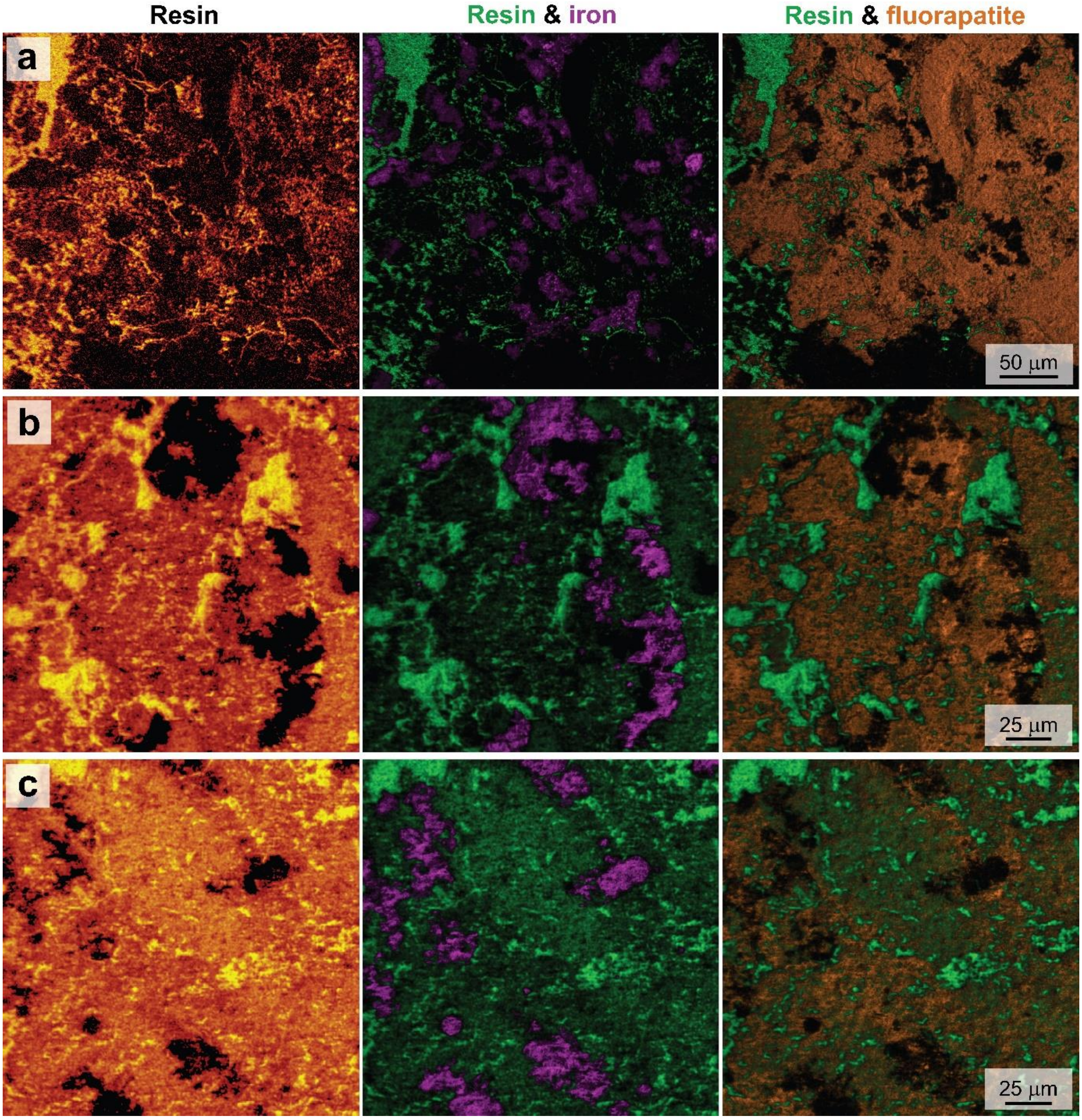

Figure S3. ToF-SIMS secondary ion maps from the coprolite across three regions (**a**, **b** and **c** as in Figure 2, 4) of the sum of resin-derived fragment ions ($C_6H_6O^+$, $C_6H_7O^+$, $C_7H_7O^+$, $C_9H_{11}O^+$). Resin maps are overlaid with secondary ion maps of iron carbonate and pyrite (middle), and fluorapatite (right) (Figure 2).

Table S1. See Supporting Information spreadsheet for this manuscript. The table presents the full sample list of Mazon Creek samples analysed by Raman spectroscopy in this study. The other 65 Phanerozoic carbonaceous metazoan fossils were previously published in Wiemann et al., 2020, with specimen details available in the Supplementary Materials of this publication. Samples are organised by fossil type with catalogue numbers, and citations for previous publication.

Table S2. Isotope values for two Mazon Creek coprolites (FMNH PE52336 from this study). All values given in permil (‰). $\delta^{34}S$ values are given for the acid volatile sulfur (AVS) and chromium reducible sulfur (CRS) fractions. *$\delta^{13}C$ values of phytane (Ph) are shown here and were previously reported in Tripp et al. (2022). Number in brackets indicates standard deviation; superscript refers to number of analyses used in average.

**Sample**			**$\delta^{34}S$**	**$\delta^{13}C$ (Ph)***
FMNH PE52316	Coprolite	AVS	9.9	-33.0 (0.11)^{2}
		CRS	8.9	
	Matrix	CRS	13.2	-29.5 (0.23)^{3}
FMNH PE52336	Coprolite	AVS	8.9	-34.7 (0.39)^{3}
		CRS	10.0	
	Matrix	CRS	7.6	-32.0 (0.31)^{3}

References

Tripp, M., Wiemann, J., Brocks, J., Mayer, P., Schwark, L., & Grice, K. (2022). Fossil Biomarkers and Biosignatures Preserved in Coprolites Reveal Carnivorous Diets in the Carboniferous Mazon Creek Ecosystem. *Biology*, *11*(9), Article 9. https://doi.org/10.3390/biology11091289

Wiemann, J., Crawford, J. M., & Briggs, D. E. G. (2020). Phylogenetic and physiological signals in metazoan fossil biomolecules. *Science Advances*, *6*(28). https://doi.org/10.1126/sciadv.aba6883

Wiemann, J., & Briggs, D. E. (2022). Raman spectroscopy is a powerful tool in molecular paleobiology: An analytical response to Alleon et al. (https://doi.org/10.1002/bies.202000295). BioEssays, 44(2), 2100070.
