# Supplementary material for: Mineralization Controls Informative Biomarker Preservation Associated With Soft Part Fossilization in Deep Time
Source: Geobiology. 2025 Sep 18;23(5):e70030. doi: 10.1111/gbi.70030 (PMC12446897; doi:10.1111/gbi.70030)
Supplement: Supplementary file 1 — Data S1: gbi70030‐sup‐0001‐supinfo.zip. [file GBI-23-e70030-s001.zip › gbi70030-sup-0002-TableS2@Table S2.docx]

**Table S2.** Isotope values for two Mazon Creek coprolites (FMNH PE52336 from this study). All values given in permil (‰). δ^34^S values are given for the acid volatile sulfur (AVS) and chromium reducible sulfur (CRS) fractions. *δ^13^C values of phytane (Ph) are shown here and were previously reported in Tripp et al. (2022). Number in brackets indicates standard deviation; superscript refers to number of analyses used in average.

**Sample δ^34^S δ^13^C (Ph)***

FMNH PE52316 Coprolite AVS 9.9 -33.0 (0.11)^2^

CRS 8.9

Matrix CRS 13.2 -29.5 (0.23)^3^

FMNH PE52336 Coprolite AVS 8.9 -34.7 (0.39)^3^

CRS 10.0

Matrix CRS 7.6 -32.0 (0.31)^3^
